# Association of known SARS-CoV-2 serostatus and adherence to personal protection measures and the impact of personal protective measures on seropositivity in a population-based cross-sectional study (MuSPAD) in Germany

**DOI:** 10.1186/s12889-023-17121-5

**Published:** 2023-11-17

**Authors:** R. Kettlitz, M. Harries, J. Ortmann, G. Krause, MuSPAD Team, Monika Strengert, Stefanie Castell, Jana-Kirstin Heise, Pilar Hernandez, Daniela Gornyk, Monike Schlüter, Tobias Kerrines, Nicole Schneiderhan-Marra, Alex Dulovic, Gerhard Bojara, Kerstin Frank, Knut Gubbe, Torsten Tonn, Oliver Kappert, Winfried V. Kern, Thomas Illig, Norman Klopp, Gottfried Roller, Michael Ziemons, Gottfried Rolle, A. Aigner, B. Lange

**Affiliations:** 1 Helmholtz Centre for Infection Research, Department Epidemiology, Brunswick, Lower Saxony Germany; 2 Translational Infrastructure Epidemiology, German Centre for Infection Research, DZIF, Düsseldorf, North Rhine-Westphalia Germany; 3 Institute for Infectious Disease Epidemiology, TWINCORE, Hannover, Lower Saxony Germany; 4 Institute of Biometry and Clinical Epidemiology, Charité-Universitätsmedizin Berlin, Corporate Member of Freie Universität Berlin and Humboldt-Universität Zu Berlin, Berlin, Berlin, Germany

**Keywords:** SARS-CoV-2, Personal protection measures, Serostatus, Germany, Non-pharmacological interventions, Seroepidemiologic studies

## Abstract

**Background:**

In 2020/2021 in Germany, several non-pharmacological interventions were introduced to lower the transmission of severe acute respiratory syndrome coronavirus 2 (SARS-CoV-2). We investigated to what extent knowledge of prior infection with SARS-CoV-2 or vaccination status influenced the use of personal protection measures (PPM). Further, we were interested in the effect of compliance with PPM on SARS-CoV-2 serostatus.

**Methods:**

Data was based on a sequential, multilocal seroprevalence study (MuSPAD), carried out in eight locations from July 2020 to August 2021. We estimated the association between a known SARS-CoV-2 serostatus (reported positive PCR test or vaccination) and self-reported PPM behavior (hand hygiene, physical distancing, wearing face mask), just as the association of PPM compliance with seropositivity against nucleocapsid (NC), receptor-binding domain (RBD), and spike protein (S) antigens. We identified relevant variables and deduced adjustment sets with directed acyclic graphs (DAG), and applied mixed logistic regression.

**Results:**

Out of the 22,297 participants (median age: 54 years, 43% male), 781 were classified as SARS-CoV-2-infected and 3,877 had a vaccinated immune response. Vaccinated individuals were less likely to keep 1.5 m distance [OR = 0.74 (95% CI: 0.57–0.97)] and only partly physically distanced [OR = 0.71 (95% CI: 0.58–0.87)]. Participants with self-reported positive PCR test had a lower chance of adhering partly to physical distancing [OR = 0.70 (95% CI: 0.50–0.99)] in comparison to the reference group. Higher odds of additionally wearing a face mask was observed in vaccinated [OR = 1.28 (95% CI: 1.08–1.51)] even if it was not obligatory. Overall, among unvaccinated participants, we found little evidence of lower odds of seropositivity given mask wearing [OR: 0.91 (95% CI: 0.71–1.16)], physical distancing [OR: 0.84 (95% CI: 0.59–1.20)] and no evidence for completely adhering to hand cleaning [OR: 0.97 (95% CI: 0.29–3.22)].

**Conclusions:**

A known confirmed prior infection and vaccination may have the potential to influence adherence to PPM.

**Supplementary Information:**

The online version contains supplementary material available at 10.1186/s12889-023-17121-5.

## Introduction

In Germany, the first SARS-CoV-2 case was registered in January 2020 [1]. The seroprevalence measured by antibodies against Spike antigens of SARS-CoV-2 in the unvaccinated population in Germany was estimated from July-December 2020 to be 1.3%–2.8% and increased in February-May 2021 to 4.1–13.1% [2].

During the pandemic and in the absence of vaccines or effective medication within the first 12 months, personal protection measures (PPM), such as physical distancing, use of face masks, adequate indoor ventilation, avoiding crowded indoor spaces, hand hygiene, and cleaning the environment were important protective measures [3, 4]. In Germany as of March 2020 lockdowns, curfews and contact bans were imposed, and various facilities, such as schools were closed and events canceled [5, 6]. The requirement of wearing face masks in public transport and for shopping was implemented on April 29, 2020, followed by lockdowns lasting from November 2020 until March 2021 [5, 6]. The vaccination campaign started at the end of December 2020 [7]. The first policy measures were eased at the beginning of March 2021, with regional differences in the opening of stores with strict hygiene measures, and citizens could get tested free of charge at test centers [5, 6].

Wearing masks, performing social distancing, having close contact for less than 15 min, and frequent hand washing were independently associated with a lower risk of a positive polymerase chain reaction (PCR) test after having had contact to a SARS-CoV-2 index case [8]. Reduced incidences of COVID-19 were associated with increased handwashing, face mask wearing, and physical distancing [9]. In addition, a combination of social distancing, testing for SARS-CoV-2, contact tracing, and quarantine could lower pressure on the health care systems [10].

In Germany, the number of newly reported infections with SARS-CoV-2 decreased between 15 to 75%, depending on the region, after the introduction of mandatory face masks, and the overall daily growth rate of reported infections decreased by 47% [11]. The requirement to wear masks in educational institutions reduced the number of infections both within and outside educational settings [11, 12].

Many of the studies performed were of ecological nature and assessed the association between recommendations or mandates and population-level infection indicators [11, 13]. The relationship between individual compliance with PPM and its impact on SARS-CoV-2 serostatus remains unclear in real-life settings. Additionally, how PPM are related to individual knowledge of vaccination and prior infection has not been assessed in larger studies – which could generate transferable knowledge to improve parametrization of future infectious disease models.

This is why we assessed participants’ behavior in the population-based study MuSPAD. The primary aim was to estimate the effect of a reported positive PCR test of SARS-CoV-2 (since the onset of the pandemic) or vaccination against SARS-CoV-2 on specific self-reported PPM behavior. The secondary aim was to evaluate the effect of self-reported adherence with PPM on seropositivity of SARS-CoV-2.

## Methods

### Study design and study population

The MuSPAD study is a German population-based seroepidemiologic cross-sectional study to assess the prevalence of antibodies against SARS-CoV-2 [2]. The data collection period was July 2020 to August 2021. The study was developed according to the WHO protocol for SARS-CoV-2 seroprevalence studies [14] and STROBE [15], and has been described before [2].

A random sample was drawn from population registration offices, stratified by age groups, gender, and spatial distribution to be representative in these aspects. Participants were 18 years or older. Included in the current analyses is a subset of the MuSPAD study comprising eight districts in Germany (Reutlingen, Freiburg, Aachen, Osnabrueck, Chemnitz, Magdeburg, Hannover (NAKO participants) and Greifswald-Vorpommern), and excluding mutations of SARS-CoV-2 and longitudinal observations. To answer the second research question, we excluded participants who were vaccinated against SARS-CoV-2 based on multiplex immunoassay (MultiCoV-Ab) [16]. Further details of the MuSPAD study were published previously [2].

## Measurements and definitions

### Specimen collection and serological testing

In each region, study centers followed standardized operating procedures when collecting blood samples. The serums were aliquoted and adequately stored at the Hannover Unified Biobank [2].

To distinguish between SARS-CoV-2 vaccinations, natural immunity after SARS-CoV-2 infection and no immunity, further analyses were performed by the MultiCoV-Ab [16]. This assay shows an improved sensitivity (88.3%) and specificity (100%) compared to other commercial tests and included analysis of using SARS-CoV-2 trimeric full-length spike protein (S), receptor-binding domain (RBD), and full-length nucleocapsid (NC) of SARS-CoV-2 and the endemic human coronaviruses [2]. A previous infection with SARS-CoV-2 was assumed before December 2020 in those with cut-offs by S > 1 and RBD > 1. From January 2021 to August 2021, the cut-off for previous infection with SARS-CoV-2 was set to S > 1, RBD > 1, and NC > 1. Vaccination against SARS-CoV-2 was based on the following values determined by the test: S > 1, RBD > 1 and NC ≤ 1. If participants were infected with SARS-CoV-2 after vaccination, they were counted as infected with SARS-CoV-2. More information is shown in the supplement (Supplement Table 2). Whether a participant had a known previous infection with SARS-CoV-2 was confirmed by a self-reported positive PCR test since the onset of the pandemic.

### Selection and measurement of variables

All relevant PPM were identified based on the recommendations of the government and health authorities [3, 4, 17]. In the analysis, we defined PPM as physical distancing, hand cleaning, and face mask wearing.

Compliance with PPM were measured via self-administered questionnaires and interviews. Physical distancing (1.5 m) and hand cleaning were assessed by the question whether participants were able to adhere “Yes, completely,” “Yes, partly” or “No, not at all”. To capture face mask compliance, participants were asked to state where and whether they wore their face masks on mandatory occasions or on mandatory plus additional occasions.

For the outcome hand cleaning, we merged the two lower categories due to the low number of observations (No, not at all: n = 91; Yes, partly: n = 6,100). We assumed that participants who do not or only partially comply with the rules do not differ too much in this context. For the outcome physical distancing, we tested each category against each other and built up three models, as we deemed all categories as distinct. For the second research question, where these variables were independent variables, we analyzed all three categories so as not to lose any information. The variable gender was included as a binary variable, distinguishing between “male” and “female” – due to a low number of observations (*n* = 4) the category “diverse” had to be excluded for the regression. More information about data preparation can be found in the supplement (Supplement data preparation).

### Statistical analysis

Descriptive statistics were used to explore the sample characteristics using median and interquartile range (IQR) for continuous variables, absolute and relative frequencies for categorical variables.

Relevant variables and their associations were determined by subject matter knowledge. Directed acyclic graphs (DAG) were used to visualize these associations and to deduce the minimal adjustment sets with the online tool DAGitty [18]. Mixed logistic regression models were applied to estimate the effect of self-reported positive SARS-CoV-2 PCR test since the onset of the pandemic or vaccination against SARS-CoV-2 on PPM compliance, just as the effect of PPM on seropositivity after an infection with SARS-CoV-2. All models included random intercepts for time and geographical region and were based on available cases. Derived odds ratio (OR) estimates were reported along with 95% confidence intervals (CI).

Data were analyzed with the statistics software R (Version 4.13), just as R packages *lme4*, *gtsummary*, and *tidyverse* [19–22].

### Ethics and data protection

MuSPAD complies with all relevant laws and declarations – EU Charter of Fundamental Rights, Biomedical Convention of the Council of Europe and additional protocols, the Council for International Organizations of Medical Sciences guidelines, and the Helsinki Declaration. Ethical approval was obtained on June 21, 2020 by the Ethics Committee of the Hannover Medical School (No 9086_BO_S_2020) and ethics committees for NAKO (Bavarian Medical Association “Bayerische Landesärztekammer” (1302313031) and Medical Association of Lower Saxony “Ärztekammer Niedersachen” (Grae/067/2013)). Furthermore, the study complies with the requirements of the General Data Protection Regulation and the Federal Data Protection Act and Recommendations for Ensuring Good Epidemiological Practice of the German Society for Epidemiology e.V. [23].

## Results

### Characteristics of the study population

The subsample included 22,927 participants from the original MuSPAD study (N = 25,712) who were eligible, offered a blood sample, and completed the PPM questionnaire. Of these, 781 were classified as infected with SARS-CoV-2, and 3,877 showed a vaccinated immune response (Table 1). Overall, the median age was 54 years (IQR = 39, 65), the same as for SARS-CoV-2-infected participants (IQR = 40, 64). Among participants vaccinated against SARS-CoV-2, the median age was 59 years (IQR = 48, 69). Of the study population, 57% were female, 60% of women were vaccinated, 41% of men were classified as naturally infected with SARS-CoV-2, and 43% had no immune response. More detailed study sample characteristics are shown below (Table 1).

**Table 1:** Demographic characteristics of study participants stratified by serological status of SARS-CoV-2

Characteristics	Overall *n* = 22,927 (%)	Serological status of SARS-CoV-2		
		Infected, not vaccinated *n* = 781(%)	No antibodies *n* = 18,269(%)	Vaccinated, no infection *n* = 3,877(%)
**Gender**				
Diverse	4 (< 0.1%)	0 (0%)	3 (< 0.1%)	1 (< 0.1%)
Female	13,167 (57%)	460 (59%)	10,363 (57%)	2,344 (60%)
Male	9,752 (43%)	321 (41%)	7,899 (43%)	1,532 (40%)
Unknown	4	0	4	0
**Age**^**a**^	54 (39, 65)	54 (40, 64)	53 (37, 64)	59 (48, 69)
Unkown	131	1	129	1
**Area**				
Rural	8,980 (39%)	368 (47%)	6,148 (34%)	2,464 (64%)
Urban	13,857 (61%)	412 (53%)	12,032 (66%)	1,413 (36%)
Unknown	90	1	89	0
**Education**				
Do not know/no information	91 (0.4%)	4 (0.5%)	80 (0.5%)	7 (0.2%)
Certificate after 9 years	2,680 (12%)	73 (9.6%)	2,252 (13%)	355 (9.2%)
Certificate after 10 years	6,758 (31%)	302 (40%)	5,216 (30%)	1,240 (32%)
Higher education certificate	12,247 (56%)	377 (50%)	9,609 (56%)	2,261 (58%)
None	90 (0.4%)	4 (0.5%)	78 (0.5%)	8 (0.2%)
Unknown	1,061	21	1,034	6
**Smoking**				
Never	11,710 (54%)	457 (60%)	9,053 (53%)	2,200 (57%)
Former	6,496 (30%)	236 (31%)	5,094 (30%)	1,166 (30%)
Occasional	948 (4.3%)	27 (3.6%)	778 (4.5%)	143 (3.7%)
Current	2,710 (12%)	40 (5.3%)	2,310 (13%)	360 (9.3%)
Unknown	1,063	21	1,034	8
**Pre-existing medical conditions**^**b**^				
0	13,188 (60%)	456 (60%)	10,629 (62%)	2,103 (54%)
≥ 1	8,661 (40%)	303 (40%)	6,594 (38%)	1,764 (46%)
Unknown	1,078	22	1,046	10
**Occupation**				
Retired / Unemployed	5,666 (26%)	194 (26%)	3,964 (23%)	1,508 (40%)
Other jobs	11,239 (52%)	350 (47%)	9,558 (57%)	1,331 (35%)
Healthcare worker, social worker, teacher	4,520 (21%)	208 (28%)	3,377 (20%)	935 (25%)
Unknown	1,502	29	1,370	103
**Household size**				
0	4,232 (19%)	114 (15%)	3,266 (19%)	852 (22%)
1	10,296 (47%)	371 (49%)	7,890 (46%)	2,035 (53%)
2	3,372 (15%)	136 (18%)	2,774 (16%)	462 (12%)
≥ 3	3,927 (18%)	134 (18%)	3,294 (19%)	499 (13%)
Unknown	1,100	26	1,045	29
**Self-reported result of PCR test since the onset of the pandemic**				
No PCR test	13,449 (62%)	202 (27%)	12,360 (72%)	887 (23%)
Tested at least once with PCR test, always negative	7,801 (36%)	176 (23%)	4,762 (28%)	2,863 (74%)
Tested at least once with PCR test, once positive	542 (2.5%)	379 (50%)	52 (0.3%)	111 (2.9%)
Unknown	1,135	24	1,095	16

^a^Median (IQR);^b^Pre-existing medical conditions: hypertension, diabetes, cancer, chronic lung disease, or immunosuppression disease

Further information of regional distribution of study sample can be found in the supplement (Supplement Table1)

### Self-reported compliance with PPM

During the observation period, 32% of participants reported full compliance with the PPM physical distancing. Stratified by serostatus, 39% of those vaccinated, 33% of those infected, and 30% of those without antibodies against SARS-CoV-2 fully adhered to physical distancing. 63% partially complied with the recommended distance of 1.5 m and 4.9% were unable to comply at all. Overall, 71% fully complied with hand cleaning, while 28% complied partly, whereby the highest compliance with hand cleaning was seen in vaccinated participants with 76%. Face masks were worn on occasions only when mandatory by 13% and on occasions when mandatory plus other occasions by 87% of the participants (Table 2). The highest compliance with face masks wearing of 88% was reported by those with no antibodies against SARS-CoV-2.

**Table 2:** Compliance with PPM of study participants stratified by serological status of SARS-CoV-2

Characteristics	Overall *n* = 22,927 (%)	Serological status of SARS-CoV-2		
		Infected, not vaccinated *n* = 781 (%)	No antibodies *n* = 18,269 (%)	Vaccinated, no infection *n* = 3,877 (%)
**Physical distance (1.5 m)**				
No, not at all	1,113 (4. 9%)	52 (6.7%)	847 (4.7%)	214 (5.5%)
Yes, partly	14,420 (63%)	466 (60%)	11,821 (65%)	2,133 (55%)
Yes, completely	7,267 (32%)	258 (33%)	5,495 (30%)	1,514 (39%)
Unknown	127	5	106	16
**Hand cleaning**				
No, not at all	91 (0.4%)	4 (0.5%)	73 (0.4%)	14 (0.4%)
Yes, partly	6,100 (28%)	206 (27%)	4,977 (29%)	917 (24%)
Yes, completely	15,356 (71%)	546 (72%)	11,895 (70%)	2,915 (76%)
Unknown	1,380	25	1,324	31
**Face mask wearing**				
Only when mandatory	2,872 (13%)	108 (14%)	2,223 (12%)	541 (14%)
Only when not mandatory	66 (0.3%)	11 (1.4%)	45 (0.2%)	10 (0.3%)
When mandatory & other occasions	19,924 (87%)	660 (85%)	15,956 (88%)	3,308 (86%)
Unknown	65	2	45	18

### Association of a self-reported positive SARS-CoV-2 PCR test and SARS-CoV-2 vaccination with PPM

To assess the relationship of SARS-CoV-2 vaccination with compliance to PPM, we deduced the following minimal adjustment sets, visualized with DAGs in Supplementary Figs. 1 and 2: age, education, gender, occupation, and pre-existing medical condition. Where known prior infection was the primary exposure, household size and smoking status were added.

**Fig. 1:**
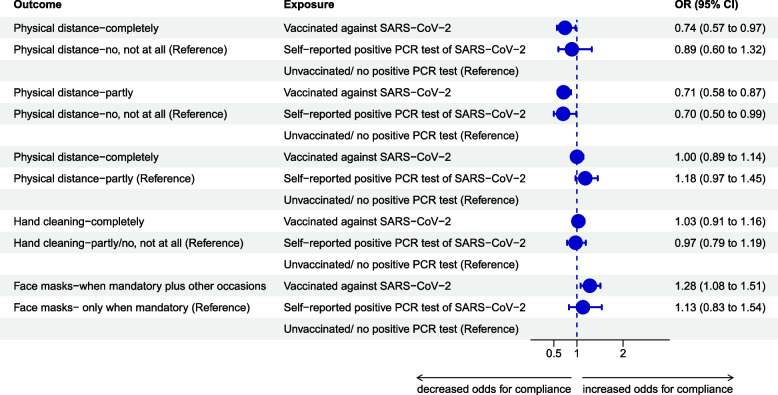
Adjusted odds ratio (OR) estimates and confidence intervals (CI) for the association of the exposures vaccination and known prior SARS-CoV-2 infection with compliance to personal protection measures

The odds of full adherence to physical distancing versus non-adherence were 26% decreased in vaccinated compared to unvaccinated participants [OR: 0.74 (95% CI: 0.57–0.97), *n* = 7,052] and 11% decreased in participants having a self-reported positive PCR test since the onset of the pandemic [OR: 0.89 (95% CI: 0.60–1.32), *n* = 7,281]. Vaccinated participants had lower odds of partial compliance to physical distancing [OR: 0.71 (95% CI: 0.58–0.87), *n* = 13,268], just as participants with a self-reported positive PCR test [OR: 0.70 (95% CI: 0.50–0.99), *n* = 13,666]. Vaccinated and unvaccinated did not relevantly differ regarding their odds of full compliance to physical distancing [OR: 1.00 (95% CI: 0.89–1.14), *n* = 18,382], but participants with a self-reported positive PCR test had higher odds to fully comply [OR: 1.18 (95% CI: 0.97–1.45), *n* = 18,917] (Fig. 1).

The odds of adhering completely to hand cleaning compared to no and partial adherence were similar in vaccinated and unvaccinated participants [OR: 1.03 (95% CI: 0.91–1.16), *n* = 18,415] and in participants having a self-reported positive PCR test compared to those who reported not having had a prior infection [OR: 0.97 (95% CI: 0.79–1.19), *n* = 18,985] (Fig. 1).

Odds of wearing face masks on mandatory plus other occasions compared to only when mandatory were 28% increased [OR: 1.28 (95% CI: 1.08–1.51), *n* = 19,353] in the vaccinated, and increased by 13% in participants having a self-reported positive PCR test compared to the reference group [OR: 1.13 (95% CI: 0.83–1.54), *n* = 19,927] (Fig. 1).

### Association of PPM with serostatus of SARS-CoV-2

Investigating the effect of PPM on serostatus in the unvaccinated, we adjusted for age, education, gender, household size, occupation, pre-existing medical condition, and smoking (Supplement Fig. 3). For the adjustment set, we identified the grade of restrictions in Germany over the course of the pandemic as causing confounding and therefore used the Health and Containment Index to account for it [24, 25].

**Fig. 2:**
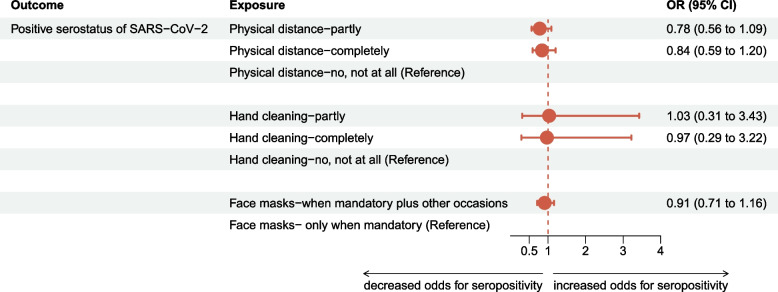
Adjusted odds ratio (OR) estimates and confidence intervals (CI) of the primary exposures compliance with personal protection measures on SARS-CoV-2 serostatus

Among the unvaccinated MuSPAD participants, the odds of being seropositive were reduced by 22% and 16%, respectively in the group that partly [OR: 0.78 (95% CI: 0.56–1.09), *n* = 15,228] and completely adhered to physical distancing [OR: 0.84 (95% CI: 0.59–1.20), *n* = 15,228] compared to non-compliance (Fig. 2).

The odds of being seropositive were similar in the group that partly [OR: 1.03 (95% CI: 0.31–3.43), *n* = 15,228] and completely [OR: 0.97 (95% CI: 0.29–3.22), *n* = 15,228] adhered to adequate hand cleaning, compared to the group reporting no compliance with hand cleaning (Fig. 2).

The odds of being seropositive in the unvaccinated participants were decreased by 9% [OR: 0.91 (95% CI: 0.71–1.16), *n* = 15,228] when wearing face masks on mandatory plus other occasions compared to those reporting wearing face masks only on mandatory occasions (Fig. 2).

## Discussion

Among MuSPAD participants in Germany the adherence to personal protection measures was rather high. Participants who were vaccinated against SARS-CoV-2 were older and had the highest compliance with physical distancing as well as hand cleaning. Those participants without an immune response against SARS-CoV-2 showed the highest percentage of additional face mask wearing. Our data indicated that self-reported positive SARS-CoV-2 PCR test since the onset of the pandemic or vaccination against SARS-CoV-2 decreased the odds of adhering more strictly to hand cleaning and physical distancing, but increased the odds of wearing face masks on mandatory occasions. In addition, we found that among unvaccinated participants, a higher adherence to PPM reduced the odds of being seropositive for SARS-CoV-2.

Overall, a high adherence to hand cleaning (67%) and additional face mask wearing (87%) was reported by the study population. In contrast, MuSPAD participants complied more partially (63%) with physical distancing. A high acceptance of PPM was also reported from an online survey (*N* = 9,796) conducted in March 2020 in various countries [26]. Researchers concluded that the prevention measures implemented by the government, for example avoiding gatherings, closure of public places, and hand hygiene were accepted by 95.0%–99.7% of the respondents [26].

Previous research has shown that being female, of older age, part of a risk group, suffering from impaired physical and mental health, having a positive perception of government communications, and mostly a higher socio-economic status were characteristics positively associated with acceptance of PPM [27–30]. In our study, we found that vaccinated participants were older and showed the highest compliance with hand cleaning and physical distancing. Further, we found that a known serostatus of antibodies against SARS-CoV-2 due to a self-reported positive PCR test or vaccination might be associated with adherence to PPM. The odds of not adhering to physical distancing were generally increased in vaccinated participants compared to those unvaccinated or not having a positive PCR test. A Study has shown that moods, such as anxiety, can enhance preventive behavior toward a SARS-CoV-2 infection [31]. Thus, a known protection with antibodies against SARS-CoV-2 could improve individuals’ moods and conversely affect compliance with PPM. Moods were not investigated in our analysis, but can be an unobserved factor, which needs to be considered in future studies.

We found that vaccinated participants and those with a known prior infection had increased odds of compliance regarding additional face mask wearing. In both exposures the odds were increased (vaccinated: OR: 1.28; self-reported positive PCR test OR: 1.13).

The extent to which PPM behavior was associated with SARS-CoV-2 seropositivity among unvaccinated MuSPAD participants was subject of the second research question. Results indicated that keeping physical distancing, hand cleaning, and wearing face masks on mandatory occasions decreased the odds of being identified as seropositive. Point estimates of the exposures of physical distancing (adhering partly OR: 0.78; adhering completely: OR: 0.84), hand cleaning (adhering partly: OR: 1.03; adhering completely: OR: 0.97), and additional face mask wearing (OR: 0.91) on seropositivity were shown to be protective. Other studies confirmed that PPM reduced the transmissions of SARS-CoV-2 [32–34]. A systematic review of 25,697 participants showed that the transmission of SARS-CoV-2 was reduced when keeping physical distance of more than 1 m [pooled adjusted OR 0.18 (95% CI: 0.09–0.38)]. Wearing face masks in addition to appropriate physical distancing can reduce SARS-CoV-2 infection by up to 85% [OR: 0.15 (95% CI: 0.07–0.34)], with a difference between respirators and disposable surgical or similar masks [33].

## Strengths and limitations

This study provided a presentation of the behavioral patterns of MuSPAD participants across Germany, based on a population level, rather than focusing only on hotspots. Data collection allowed depicting PPM adherence across Germany and at different times. Another strength was the measurement of SARS-CoV-2 serostatus using multiplex immunoassay to distinguish between vaccinated, recovered, and individuals without antibodies against SARS-CoV-2. Compared with commercial assays, this test already demonstrated its improved sensitivity [16] in comparison to case identification based on (self-reported) PCR tests used in previous studies [8]. To capture relevant confounding, adjustment sets were derived from expertise, visualized with DAGs, and evaluated jointly in the research group.

Due to the sequential cross-sectional nature of the MuSPAD, the temporal sequence of the relationship between exposure and outcome is uncertain. In addition, response bias due to self-report questionnaires may result in overestimation [35]. Due to mandatory mask wearing, social desirability is an important factor that may influence our estimates [36], too. The Health and Containment Index to control for restrictions in Germany [25] was only available on a country and not on a communal level, limiting the accuracy of adjustment.

Another aspect that needs to be discussed is that the large overall sample size of our study may result in confidence intervals being very narrow, suggesting high precision in the effect estimates, resulting in more statistically significant findings. Therefore, in the interpretation of our findings we focused on precision estimated based on confidence intervals.

In addition, we would like to point out that for each outcome and exposure, a specific regression model was built to adequately control for confounding factors for each research question. Therefore, there is a possibility that some of our results are false positives due to multiple testing. In our questionnaire, compliance was measured in three categories (“No, not at all,” “Yes, partly,” “Yes, completely”). Therefore misclassification might have occurred, as adherence to PPM could not be captured accurately and thus differences between individuals could not be validly measured. In our categorization of vaccinated and infected due to serological status we did not account for those who were both, which potentially overestimated the association between personal protective measures and infections according to serological status. However for the time period assessed here this misclassification cannot be large as the proportion of those infected before vaccination was small.

## Conclusion

A prior known infection of SARS-CoV-2 confirmed with a positive PCR test and vaccination against SARS-CoV-2 may have influenced compliance with certain PPM behaviors during the pandemic in Germany. Furthermore, similar to previous research, we confirmed that compliance to PPM can reduce the odds of SARS-CoV-2 seropositivity in unvaccinated participants.

### Supplementary Information

**Additional file 1.** DAG - variable Selection of adjustment set. Data preparation regression models. Regional distribution of study sample. Self-reported vaccination status and self-reported result of PCR test and by serostatus.

## Data Availability

The anonymized data for this study will be made available to other academic researchers. To request additional data or information from this study, please contact the author Manuela Harries (Manule.Harries@helmholtz-hzi.de). Institutions can apply for the data via serohub@helmholtz-hzi.de. Regarding data from the NAKO study Hannover researchers have the opportunity to apply for data usage in accordance with the official regulations and specifications. For more detailed information, please visit https://transfer.nako.de.

## Abbreviations

CDC: Centers for Disease Control and Prevention
CI: Confidence interval
COVID-19: Coronavirus disease 2019
DAG: Directed acyclic graph
ECDC: European Centre for Disease Prevention and Control
HUB: Hannover Unified Biobank
HZI: Helmholtz Centre for Infection Research
MultiCoV-Ab: Multiplex immunoassay
MuSPAD: Multilocal and Serial Prevalence Study of Antibodies to SARS-2 coronavirus in Germany
NC: Nucleocapsid
NMI: Natural and Medical Sciences Institute at the University of Tübingen
OR: Odds ratio
PCR: Polymerase chain reaction
PPM: Personal protection measures
RBD: Receptor-binding domain
RKI: Robert Koch Institute
RNA: Ribonucleic acid
S: Spike protein
SARS-CoV-2: Severe acute respiratory syndrome coronavirus 2
WHO: World Health Organization
