# Publisher Correction to: Association of known SARS-CoV-2 serostatus and adherence to personal protection measures and the impact of personal protective measures on seropositivity in a population-based cross-sectional study (MuSPAD) in Germany

**DOI:** 10.1186/s12889-024-18125-5

**Published:** 2024-02-23

**Authors:** R. Kettlitz, M. Harries, J. Ortmann, G. Krause, MuSPAD Team, A. Aigner, B. Lange

**Affiliations:** 1 Helmholtz Centre for Infection Research, Department Epidemiology, Brunswick, Lower Saxony Germany; 2 Translational Infrastructure Epidemiology, German Centre for Infection Research, DZIF, Dusseldorf, North Rhine‑Westphalia Germany; 3 Institute for Infectious Disease Epidemiology, TWINCORE, Hannover, Lower Saxony Germany; 4 Institute of Biometry and Clinical Epidemiology, Charite-Universitatsmedizin Berlin, Corporate Member of Freie Universitat Berlin and Humboldt-Universitat Zu Berlin, Berlin, Germany

**Publisher Correction to: BMC Public Health 23, 2281 (2023)**

**https://doi.org/10.1186/s12889-023-17121-5**

Due to an error in the publication [1] process reference 6 was split into 2 separate references: 6 and 7. This caused all citations to be incorrect from 7 onwards. The incorrect references 6 & 7 are listed below along with the correct reference 6. The original article has been updated to correct these errors. The publisher apologizes to the authors & readers for the inconvenience caused by this.

**Incorrect**

6. Bundesgesundheitsministeriums. Coronavirus-Pandemie: Was geschah wann?

7. Chronik aller Entwicklungen im Kampf gegen COVID-19 (Coronavirus SARS-CoV-2) und der dazugehörigen Maßnahmen des Bundesgesundheitsministeriums 2022 [Available from: https://www.bundesgesundheitsministerium.de/coronavirus/chronik-coronavirus.html.

**Correct**

6. Bundesgesundheitsministeriums. Coronavirus-Pandemie: Was geschah wann? Chronik aller Entwicklungen im Kampf gegen COVID-19 (Coronavirus SARS-CoV-2) und der dazugehörigen Maßnahmen des Bundesgesundheitsministeriums 2022 [Available from: https://www.bundesgesundheitsministerium.de/coronavirus/chronik-coronavirus.html.
