# Supplementary material for: Association of known SARS-CoV-2 serostatus and adherence to personal protection measures and the impact of personal protective measures on seropositivity in a population-based cross-sectional study (MuSPAD) in Germany
Source: BMC Public Health. 2023 Nov 17;23:2281. doi: 10.1186/s12889-023-17121-5 (PMC10657116; doi:10.1186/s12889-023-17121-5)
Supplement: Supplementary file 1 — Additional file 1. DAG - variable Selection of adjustment set. Data preparation regression models. Regional distribution of study sample. Self-reported vaccination status and self-reported result of PCR test and by serostatus. [file 12889_2023_17121_MOESM1_ESM.docx]

# Supplement

### 1 DAG - variable Selection of adjustment set

#### DAG: Association of vaccination on compliance with PPM


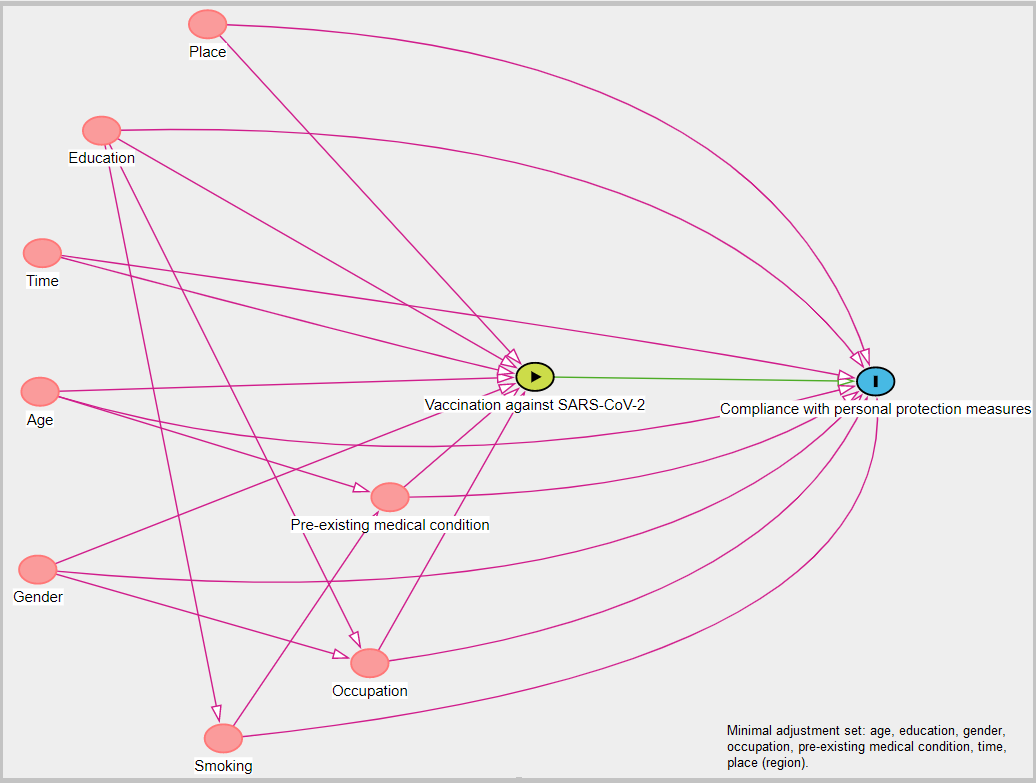


Supplement Figure 1: Directed acyclic graph: Association of vaccination on compliance with PPM and designed by using the online tool DAGitty (18).

*1.2 DAG: Association of suspected or confirmed prior infection with SARS-CoV-2 on compliance with PPM*


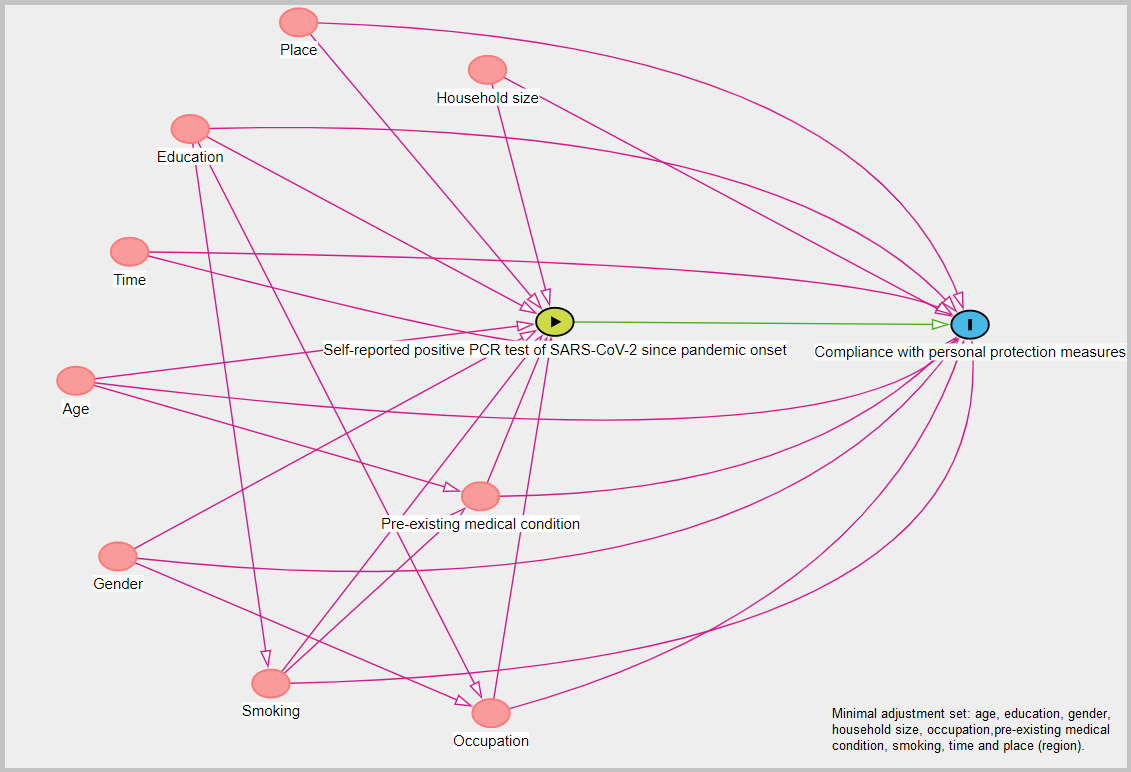


Supplement Figure 2:Directed acyclic graph: Association of suspected or confirmed prior infection with SARS-CoV-2 on compliance with PPM and designed by using the online tool DAGitty (18).

#### 1.3 DAG: Association of PPM on natural seropositivity after SARS-CoV-2 infection


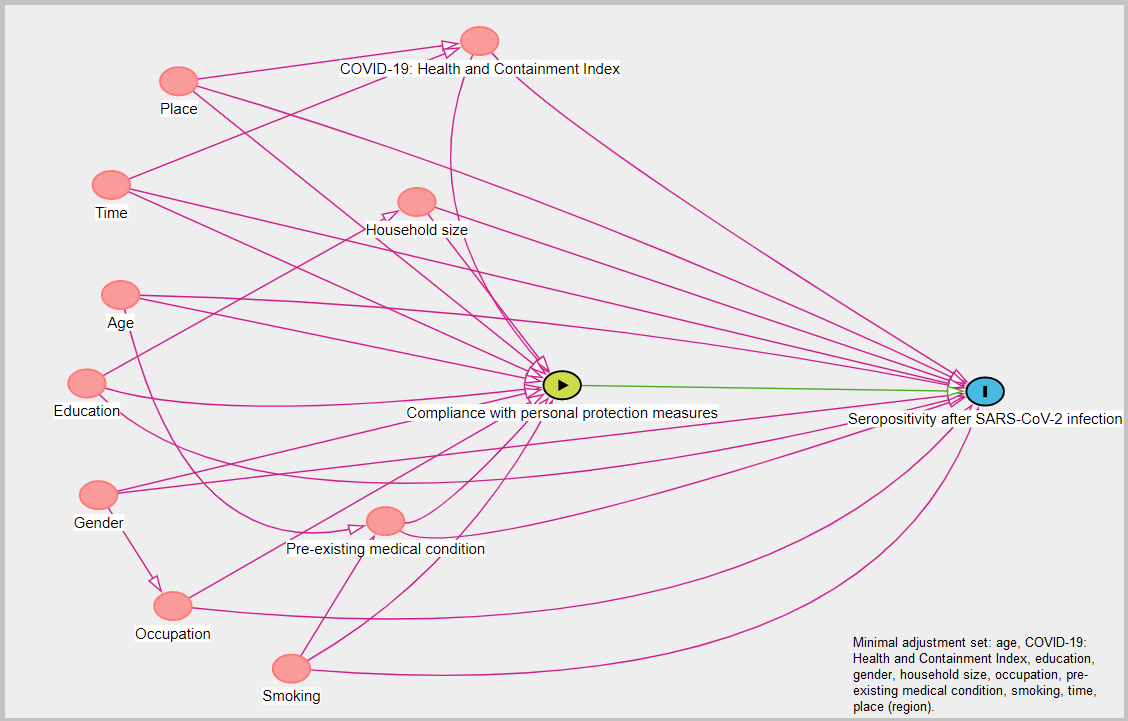


Supplement Figure 3: Directed acyclic graph: Association of PPM on natural seropositivity after infection with SARS-CoV-2 and designed by using the online tool DAGitty (18).

## *2 Data preparation regression models*

When hand cleaning was set to be the dependent variable, the category “No, not at all” of hand cleaning was merged with the category “Yes, partly,” due to the fact that there were only 91 observations and our assumptions that the lowest category was close to the middle category. For the variable physical distancing as the dependent variable, we considered all categories and tested each category against each other. The variable mask wearing distinguished between wearing face masks on occasions when it was only mandatory (reference category) and on occasions where mask wearing were set to be mandatory plus other occasions. The exposure variables of vaccination against SARS-CoV-2 (reference category= “not vaccinated”) and known prior infection with SARS-CoV-2 (self-reported positive PCR SARS-CoV-2 test since the start of the pandemic, no positive PCR test as reference category) were implemented as categorical variables.

For the second research question, the outcome variable was coded as seropositive after infections with SARS-CoV-2 based on the multiplex immunoassay and the reference category where participants showed no antibodies against SARS-CoV-2. The exposures wearing face masks were coded as above. Hand cleaning and physical distancing were included with all three categories.

Other relevant variables were measured as follows: age was a numeric variable in years. The variable gender was included as a binary variable, distinguishing between male and female. The category diverse was excluded for the regression analysis due to the low number of observation (n=4). The variable education (reference category = “certificate after 9 years;” 1 = “certificate after 10 years;” 2 = “higher education certificate”). The lowest category “no certificate” was merged to the reference category due to the small number of observations in the analytical part of the study. The variable occupation distinguished between certain groups of occupation and being similar in their working environment related to being exposed to SARS-CoV-2, such as “healthcare worker, social worker, teacher,” “retired participants and no employment,” or other jobs.

Pre-existing medical conditions (reference category = ”no pre-existing medical conditions;” category of interest = “one or more pre-existing medical conditions”), smoking (reference category=never, 1=former, 2=occasional, 3=current), and household size (categories ranged from living alone as reference category, 1=plus one person, 2=plus two persons, 3=plus three or more additional persons living in one household), and the Health and Containment Index (“50–59,” “60–69,” “≥70”) were also considered as categorical variables.

### 3 Regional distribution of study sample

Supplement Table 1: Regional distribution of study participants

| **Characteristics** | **Overall, N = 22,927** |
| --- | --- |
| **Region** | **Median (IQR) or Frequency (%)** |
| Reutlingen (July 2020) | 1,656 (7.2%) |
| Freiburg (August–September 2020) | 2,143 (9.3%) |
| Aachen (September–October 2020) | 1,053 (4.6%) |
| Osnabrueck (October–November 2020) | 2,474 (11%) |
| Reutlingen ((October–November 2020) | 2,015 (8.8%) |
| Magdeburg (November–December 2020) | 1,941 (8.5%) |
| Freiburg (November–December 2020) | 1,476 (6.4%) |
| Aachen (January–February 2021) | 1,227 (5.4%) |
| Chemnitz (March 2021) | 2,005 (8.7%) |
| Osnabrueck (March 2021) | 1,182 (5.2%) |
| Magdeburg (April–May 2021) | 1,016 (4.4%) |
| Vorpommern-Greifswald (May–June 2021) | 1,683 (7.3%) |
| Hannover (June–July 2021) | 2,212 (9.6%) |
| Chemnitz (July–August 2021) | 844 (3.7%) |

### 4 Self-reported vaccination status and self-reported result of PCR test and by serostatus

Supplement Table 2: Self-reported vaccination status and self-reported result of PCR test and by serostatus

| **Characteristics** | **Overall**  **n = 22,927 (%)** |  | **Serological status of SARS-CoV-2** |  |
| --- | --- | --- | --- | --- |
|  |  | Infected, not vaccinated  n = 781 (%) | No antibodies  n = 18,269 (%) | Vaccinated, no infection  n = 3,877 (%) |
| **Self-reported first dose vaccination of SARS-CoV-2** | 4,320 (19%) | 113 (15%) | 530 (2.9%) | 3,677 (95%) |
| Unknown | 137 | 8 | 121 | 8 |
| **Self-reported second dose vaccination of SARS-CoV-2** | 2,346 (14%) | 51 (15%) | 47 (0.4%) | 2,248 (61%) |
| Unknown | 5,969 | 431 | 5,320 | 218 |
| **Self-reported result of PCR test** |  |  |  |  |
| No PCR test | 13,449 (62%) | 202 (27%) | 12,360 (72%) | 887 (23%) |
| Tested at least once with PCR test, always negative | 7,801 (36%) | 176 (23%) | 4,762 (28%) | 2,863 (74%) |
| Tested at least once with PCR test, once positive | 542 (2.5%) | 379 (50%) | 52 (0.3%) | 111 (2.9%) |
| Unknown | 1,135 | 24 | 1,095 | 16 |
|  | | | | |
